# Criteria for developing, assessing and selecting candidate EQ-5D bolt-ons

**DOI:** 10.1007/s11136-022-03138-7

**Published:** 2022-04-29

**Authors:** Brendan J. Mulhern, Chris Sampson, Phil Haywood, Rebecca Addo, Katie Page, David Mott, Koonal Shah, Mathieu F. Janssen, Mike Herdman

**Affiliations:** 1 Centre for Health Economics Research and Evaluation, University of Technology Sydney, Sydney, Australia; 2 Office of Health Economics, London, UK; 3 PHMR, London, UK; 4 National Institute for Health and Care Excellence, London, UK; 5 EuroQol Research Foundation, Rotterdam, Netherlands

**Keywords:** EQ-5D, Bolt-ons, Psychometrics, Health-related quality of life

## Abstract

**Purpose:**

‘Bolt-on’ dimensions are additional items added to multi-attribute utility instruments (MAUIs) such as EQ-5D that measure constructs not included in the core descriptive system. The use of bolt-ons has been proposed to improve the content validity and responsiveness of the descriptive system in certain settings and health conditions. EQ-5D bolt-ons serve a particular purpose and thus satisfy a certain set of criteria. The aim of this paper is to propose a set of criteria to guide the development, assessment and selection of candidate bolt-on descriptors.

**Methods:**

Criteria were developed using an iterative approach. First, existing criteria were identified from the literature including those used to guide the development of MAUIs, the COSMIN checklist and reviews of existing bolt-ons. Second, processes used to develop bolt-ons based on qualitative and quantitative approaches were considered. The information from these two stages was formalised into draft development and selection criteria. These were reviewed by the project team and iteratively refined.

**Results:**

Overall, 23 criteria for the development, assessment and selection of candidate bolt-ons were formulated. Development criteria focused on issues relating to i) structure, ii) language, and iii) consistency with the existing EQ-5D dimension structure. Assessment and selection criteria focused on face and content validity and classical psychometric indicators.

**Conclusion:**

The criteria generated can be used to guide the development of bolt-ons across different health areas. They can also be used to assess existing bolt-ons, and inform their inclusion in studies and patient groups where the EQ-5D may lack content validity.

## Plain English summary

It is important to accurately measure patient’s health and quality of life to ensure that all areas of health of relevance to particular conditions are included. This means that health decision making is informed by valid patient responses. However, many questionnaires do not cover all constructs, and for some, such as the widely used EQ-5D questionnaire, additional questions can be developed. There is no published guidance available about how to develop those questions. The aim of this paper is to outline a set of guidance criteria for the development and selection of new questions for existing questionnaires.

## Introduction

The EQ-5D is the most widely used multi-attribute utility instrument (MAUI) of health-related quality of life (HRQoL) internationally [1]. The descriptive system includes five dimensions of health: mobility, self-care, usual activities, pain/discomfort and anxiety/depression). However, there is a growing body of evidence suggesting that, in some circumstances, the EQ-5D descriptive system may not be sensitive to the health impacts of certain conditions. For example, mixed-methods research has found limitations in the validity of the EQ-5D in severe mental health conditions [2, 3] and vision and hearing problems [4]. Therefore, changes in HRQoL that are considered important in these conditions may not be detected. This has implications for the sensitivity and validity of the EQ-5D in resource allocation decision making. Qualitative evidence also suggests that members of the public perceive the EQ-5D descriptive system to be missing important aspects of health, particularly with respect to sensory deprivation and mental health, and identify vision/sight and cognition/mental functioning when asked to list aspects of health that they consider important [5].

In response to concerns around the measurement limitations of the EQ-5D descriptive system, there has been interest in developing ‘bolt-ons’ for the EQ-5D. Box 1 explains the terminology used to describe the different features of bolt-ons in this paper. Bolt-ons add dimensions of health to the EQ-5D in situations where they may improve its coverage, sensitivity and responsiveness to change over time [4, 6]. A recent review of methods used to develop bolt-ons is available [7]. The review paper identified 26 bolt-ons for EQ-5D and found that a wide variety of bolt-on identification methods, psychometric performance tests and health state valuation methods were used in the included studies. Many of the candidate bolt-ons developed to date relate to generic functional health constructs. This means they can be used across different health conditions where an impact on the construct being measured is expected. Examples of these include bolt-ons to measure sleep, hearing, vision, cognition, respiratory problems and energy [4, 8–13]. Nominally condition-specific bolt-ons have also been developed, for example to measure the impacts of psoriasis [14].

Box 1: Terminology used to describe bolt-ons

**Table:** 

**Bolt-on**: One or more items to collectively describe a single health problem or condition being added to an existing instrument **Item**: a single question asking about a dimension or domain, or functional impairment **Descriptor**: any response level or other part of the phrasing used in the description of an item **Title:** The overall title of each EQ-5D item that appears above the descriptor
**Dimension:** the underlying concepts that bolt-on items and descriptors are intended to describe

Although there has been substantial work developing and testing bolt-ons, there are no widely accepted bolt-on descriptors. For instance, the EuroQol Research Foundation’s suite of instruments does not include any fully approved bolt-ons (https://euroqol.org/eq-5d-instruments). To date, there has been limited work to establish a core set of methods that could be consistently applied to develop bolt-ons in any health area, and guidelines to standardise the wording of the bolt-on dimension and response options, and facilitate quality assessment, have been called for [7]. EQ-5D bolt-on descriptors need to serve a particular purpose and thus satisfy certain criteria. There are at least two respects in which the development of a bolt-on must differ from the development of a novel instrument. First, bolt-on descriptors must be suitable for presentation alongside the core set of existing descriptors. Second, the performance of a bolt-on descriptor must be judged in combination with the existing descriptors at the item level to understand how the additional descriptor adds to the information collected by the core descriptive system. For these reasons, it is likely that the criteria that a bolt-on descriptor should satisfy will be distinct from that for other novel MAUIs. To date, no such criteria have been defined. Therefore, the aim of this paper is to generate a set of generic criteria to guide the development, assessment and selection of candidate bolt-ons to EQ-5D. These criteria are not developed to be prescriptive, but can be used to inform the selection of bolt-on descriptors to recommend for further use. They can also be used to inform the assessment of the development process and properties of existing bolt-ons.

## Methods—development of the criteria

The development of the criteria was based on an iterative approach. The initial criteria were informed by prior bolt-on work [7], best practice and experience, earlier criteria used for the development of the SF-6Dv2 classification system [15] and the COnsensus-based Standards for the selection of health status Measurement Instruments (COSMIN) checklist [16]. The relevance criteria for broader preference-based measure item development that were published during the development of the criteria reported in this paper [17] was also considered in the context of bolt-on dimensions. The development of the criteria was based on retaining the advantages of the EQ-5D, specifically the brevity and the minimal burden of completion. Also considered was the importance of consistency, incorporating qualitative information from people with lived experience, and incorporating quantitative data related to the psychometric properties of bolt-ons. Throughout the development of the criteria, we also considered valuation-related issues, with the aim to ensure that the criteria would lead to bolt-ons that were amenable to valuation using widely accepted methods (such as time trade-off and discrete choice experiments).

**Fig. 1:**
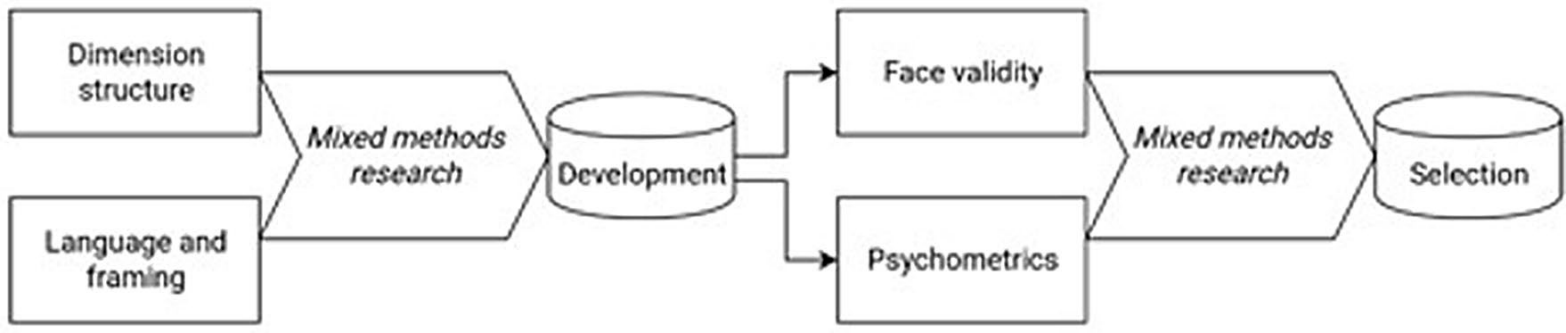
Bolt-on development and selection process

The criteria were divided into two main groups based on the bolt-on development and selection process developed by the author team (Fig. 1.). The first group consisted of development criteria that would be used to generate candidate bolt-ons. The second group consisted of selection criteria that would be used to compare and choose between different candidate bolt-ons.

Mixed methods were considered for the criteria: the combination of both qualitative and quantitative research is necessary for the selection of meaningful descriptors and to allow for a clear understanding of the implications of their use as bolt-ons. It is recommended that the development of health state descriptors for MAUIs should be informed by qualitative research [18, 19]. Quantitative analysis can then assess the measurement properties of the bolt-ons developed.

A draft set of criteria was developed by a subset of authors (BJM, PH, RA and KP), informed by a review of existing measures and criteria for their development and qualitative work conducted by the wider team for vision and cognition bolt-on development. These were then presented to the remainder of the project team and revised in accordance with their input. The draft criteria were also presented to a group of health economists at the Centre for Health Economics Research and Evaluation, University of Technology Sydney, and at meetings of the EuroQol Group (including an early career researcher conference and meetings of the Descriptive System Working Group).

The criteria have been developed as part of an ongoing project to develop bolt-on dimensions for vision and cognition using a structured qualitative and quantitative approach [20]. The draft criteria were also considered during the literature review and focus group stages of that larger project. This led to further refinement and development of additional criteria.

## Results

Overall, 23 criteria were developed. These were divided into two groups focused on development and selection of bolt-ons. The criteria in each group are described below.

### Bolt-on development criteria

There were two subgroups to the bolt-on development criteria group. The first focused on dimension structure, and the second on dimension language and framing. These criteria emphasised consistency with the core EQ-5D descriptive system and relevance to the condition/HRQoL construct for which the bolt-on is being developed. Table 1 reports five criteria focused on dimension structure. For each criteria, the reasoning behind the criteria and potential issues are also explained. A key focus of these criteria is around consistency with the existing EQ-5D dimensions structure, including consistency with the dimension title format (Criteria 1–2), and response levels (Criteria 3–4). This promotes ease of completion, parsimony and amenability to valuation (by simplifying the health state descriptions required for valuation). For example, Criteria 2 focuses on the dimension title and supports consistency by specifying examples of a particular construct in parentheses. This is in line with usual activities in the EQ-5D, which specifies work, study, housework, family or leisure activities as examples. Possible issues these criteria raise are that the use of examples/descriptions could lack cross-cultural validity if not universal, and they limit the applicability of single bolt-on for both the EQ-5D-5L and EQ-5D-3L. Complex sentences may also be possible. For example, using longer descriptions of functioning problems, or framing as positive or negative constructs, can complicate the item structure.

**Table 1:** Bolt-on development criteria for EQ-5D dimension structure

Number	Criteria	Reasoning for criteria	Potential issues with criteria
1	Short dimension title	Consistency with existing dimension titles	May not be possible for some condition-specific bolt-ons
2	Dimension title examples and explanations in parenthesis (if necessary)	Consistency and parsimony with existing dimensions. Increased ease of comprehension and completion	Use of examples/descriptions could lack cross cultural validity if not universal
3	Number of response levels should be consistent with the EQ-5D version for which the bolt-on is being developed	Consistency with existing structure, and ease of completion and valuation	Applicability of single bolt-on for both EQ-5D-5L and EQ-5D-3L
4	Response levels formed as sentences	Consistency with existing structure more likely to be acceptable, ease of completion	Complex sentences possible
5	Minimal additional number of items per bolt-on	Parsimony with approach used for other EQ-5D dimensions, and amenability to valuation	One item might not capture a substantial portion of the clinical condition in certain contexts. There may be a case for numerous items for complex conditions

Table 2 reports six criteria focused on dimension language and framing. These are focused on developing brief and concise generic descriptors (Criteria 6 and 8) that are widely translatable in terms of language and culture (Criteria 7). Regarding framing, it is specified that dimension wording should be the same as the core EQ-5D dimensions (Criteria 9), and response levels should be framed as severity where possible (Criteria 10). Finally, Criteria 11 specifies that the language used should be informed by qualitative work with relevant patient groups and populations (Criteria 9). This set of criteria is important to increase international applicability of the bolt-on, promote consistency with the dimension descriptions used in the current EQ-5D, and improve relevance to patient groups to increase validity.

**Table 2:** Bolt-on development criteria for EQ-5D dimension language and framing

Number	Criteria	Reasoning for criteria	Potential issues with criteria
6	Brief and concise bolt-on descriptors	Consistency and parsimony of bolt-on presentation	Short descriptions may not be fully informative about the construct measured
7	Bolt-on descriptors should be translatable (in terms of language, and cross culturally in countries where the bolt-on is applicable)	Increase international applicability of the bolt-on	International work may be beyond the scope of the project
8	Generic descriptions preferred to condition-specific descriptions	Consistency with descriptions used in current EQ-5D. Generic descriptions can be used across different health conditions where an impact on the construct being measured is expected	Items may lack precision or salience for some conditions. Specific examples allow for more sensitive and focused measurement
9	Bolt-on descriptors informed by language used in qualitative work (e.g. focus groups)	Relevance to patients increases validity. Allows for use of primary qualitative data in the development process	Language preferred may not fit in the context of EQ-5D bolt-ons, or be translatable cross culturally
10	The direction of dimension wording should be the same as the core EQ-5D dimensions (i.e. negative)	Consistency with existing EQ-5D improves completion, avoids psychometric effects linked to response level wording, and increases amenability to valuation	Different framing of the response level wording may be more applicable to a particular bolt-on. May conflict with narratives adopted by people with disabilities
11	Response options framed as severity preferred to other possible framings (e.g. frequency)	Consistency with existing EQ-5D improves completion, avoids psychometric effects linked to inconsistencies, and increases amenability to valuation	Different framing of the response options may be more applicable to a particular bolt-on. May contradict how people with lived experience think about their health state

### Bolt-on assessment and selection criteria

The criteria in this group focused on bolt-on selection criteria based on assessing the face and content validity and psychometric performance of the candidate bolt-ons. Table 3 presents four face and content validity criteria. These are specified to ensure that the dimension and response level wording is comprehensible and can be completed (Criteria 12 to 14), and the bolt-ons have content validity across patient/population groups with different but relevant health problems and severity of problems (Criteria 15). Potential issues with these criteria are that they may be difficult to assess in all populations for which the bolt-on is potentially relevant.

**Table 3:** Bolt-on selection criteria for face and content validity

Number	Criteria	Reasoning for criteria	Potential issues with criteria
12	Dimensions are worded in a comprehensible way	Supports ease of completion, and means item wording is relevant to patients	May be difficult to test in all relevant populations
13	Severity levels descriptors are comprehensible and salient	Supports ease of completion, and means item wording is relevant to patients	
14	Concepts included are understandable, relevant and relatable	Supports ease of completion, and means item wording is relevant to patients	
15	Bolt-ons have content validity across groups with different but relevant health problems and severity of problems	Ensure sensitivity and relevance of items to different populations and condition severities	

Table 4 describes seven criteria linked to classical psychometric assessment methods. These criteria are important in quantitatively assessing the characteristics of the bolt-on, to support the final selection of bolt-ons to recommend for use. The classical psychometric criteria focus on a range of established tests including acceptability in terms of response patterns (Criteria 16 to 18), to ensure that all levels are endorsed, and relevant, and there is not strong evidence of a ceiling effect. Issues with these criteria may be linked to the existence of subgroup specific response patterns that may not reflect the overall population for which the bolt-on is relevant. Criteria 19 focuses on the psychometric property of reliability, namely test–retest reliability, which ensures that responses are stable over time, where change in response to the bolt-on is not expected.

**Table 4:** Bolt-on selection criteria for psychometrics

Number	Criteria for assessing candidate bolt-on	Reasoning for criteria	Potential issues with criteria
16	Acceptability—Spread of item responses across categories (aggregate adjacent frequencies > 10%, and maximum endorsement frequencies < 80%^a^	Demonstrates that all levels are endorsed, and are therefore relevant	Might be population specific response patterns
17	Acceptability – missing data < 5%^a^	Missing data indicates possible issues with responding to the item. Increases likelihood of full completes for use in analysis	Item could be acceptable to certain conditions, but not others, who may display more missing data
18	Acceptability—Level 1 (no problems) category use < 80%^a^	Limits extent of ceiling effects alongside core EQ-5D dimensions. This is important as a high ceiling effect limits the item’s sensitivity to improvements in the construct	Might be population specific response patterns
19	Reliability (test–retest) – Moderate or high Intraclass Correlation Coefficient (< 0.8)^a^	Ensures stability of responses to the bolt-on over time (where change is not expected to occur)	Data to allow for assessment of test–retest reliability may not be commonly available
20	Construct validity – Bolt-on diverges (low correlations < 0.4) with core EQ-5D dimensions (where divergence expected)^b^	Demonstrates that what is being measured differs to the core dimensions (to different extents)	Level of expected divergence is unknown, so is inferred
21	Construct validity (convergence) –Bolt-on has construct validity (moderate (> 0.3 or large correlations > 0.7) with existing dimensions (where overlap is expected) and other relevant measures of overlapping HRQoL constructs^b^	Demonstrates relationship with existing measures developed specifically for particular conditions, and existing EQ-5D dimensions (when expected)	Level of expected convergence is unknown, so is inferred
22	Construct validity (known group) – Bolt-on has evidence of sensitivity (moderate > 0.5) to large (> 0.8) effect sizes or significant differences) to subsamples (where a difference in response patterns may be expected)^b^	Demonstrates that bolt-on is sensitive to populations with different levels of an existing trait (for example different levels of quality of life impairment)	Known group validity may be more challenging to assess based on single bolt-ons rather than longer scales
23	Responsiveness – Bolt-on is responsive to change in the HRQoL concept measured by the bolt-on over time (moderate > 0.5) to large (> 0.8) effect sizes or significant difference^b^	Demonstrates that bolt-on is sensitive to improvement and decreases in health status over time	Data to allow for assessment of bolt-on responsiveness may not be commonly available. Change assessment based on single bolt-ons rather than longer scales

^a^Criteria based on previous work assessing item acceptability in scale development [21, 22]

^b^criteria based on guidelines specified for correlations and effect sizes by Cohen [23]

Criteria 20 to 22 focus on assessing elements of construct validity to demonstrate that what is being measured differs to the core dimensions (to different extents), but has a relationship with existing measures developed specifically for similar or overlapping health condition, and can detect known differences when expected. The criteria for examining the extent of the evidence for construct validity are based on established cut off points for correlations and effect sizes [21–23]. These analyses may be more challenging for single bolt-ons, and the level of the expected relationship is unknown, so it must be inferred. Criteria 23 focuses on guidance around assessing responsiveness to change to demonstrate that the bolt-on is sensitive to improvement and decreases in the HRQoL construct measured by the bolt-on over time. However, data to allow for assessment of bolt-on responsiveness may not be commonly available.

## Discussion

This paper outlines a set of criteria to provide guidance for the development of EQ-5D bolt-ons and assessment of their relative performance. These can be used to guide the development and selection of future bolt-ons, and the assessment of existing bolt-ons, increasing the transparency and validity of bolt-on work. The contribution of this paper is to make the criteria underlying bolt-on development and assessment processes and decisions transparent and thus aid further development and reproducibility. We also identify some of the consequences and trade-offs that may occur in the development of bolt-ons.

Our proposed criteria are not necessarily prescriptive, but rather make plain the decisions and trade-offs required in the development of bolt-ons. A noteworthy lesson from our work is that a trade-off must be made in ‘language and framing,’ between the terminology preferred by people with lived experience and consistency with existing EQ-5D descriptors. As such, the development of any bolt-on is constrained by existing parameters and measurement issues inherent in the base measure. This has both strengths and weaknesses. Consistency with existing EQ-5D descriptors avoids psychometric effects linked to response level wording and increases amenability to valuation. The same reasoning applies to the dimension structure and face validity testing of selected bolt-on items. However, gaining consistency and ease of valuation comes at the expense of arguably the most accurate reflection of the patient voice and a greater depth of understanding (due to limiting the number of items). Such trade-offs are inevitable in the development of any measure, particularly preference-based measures.

Our study suggests criteria in line with the original intent of bolt-ons: to complement existing EQ-5D instruments rather than develop new measures for a particular condition under consideration. Our work complements other published criteria supporting the development of preference–based instruments [17] and fulfils a recommendation for guidelines for bolt-on development by a recent assessment of the methods used to develop bolt-ons [7]

The specific requirements of bolt-ons meant that a number of areas of commonly used psychometric assessment methods were not included, or may be challenging to conduct. First, this included measures of reliability assessment beyond test–retest, such as internal consistency. This evaluates if the domains of an instrument are measuring the same construct, it is therefore not relevant for bolt-ons given the use of single item dimensions. Second, we did not include Item Response Theory methods [21] that are a set of generalised linear models that link observed item responses to respondents’ location on an unmeasured underlying latent trait and have gained prominence in the development and testing of patient-reported outcome measures. An issue with these criteria is the general requirement for unidimensionality of multiple item domains which would mean IRT would be conducted by comparing bolt-ons to items measuring similar or overlapping constructs from other instruments. Although this approach can be used to assess bolt-on performance, the interpretation of the results in comparison with domains from other instruments is too complex for inclusion in a set of general guidance criteria. Therefore we focused on criteria linked to classical psychometric tests. We also note that the psychometric criteria could be limited by the data available, and meeting the criteria may not always be possible. For example, construct validity requires valid comparator measures, and test–retest and responsiveness assessment require longitudinal data. However, we encourage developers of bolt-ons to design validation studies to allow for psychometric assessments to be conducted.

Although this paper focuses on criteria for the development of bolt-ons for EQ-5D, the three overall criteria categories, and individual criteria, could also provide guidance for the development of bolt-on dimensions for other MAUIs. For example, in the development of HRQL items, the structure of the items and the language and framing used are key considerations. Using face and content validity approaches to examine and select items is a key stage of instrument development, as is assessment of performance using psychometric methods. The individual criteria can be considered in reference to the instrument that additional items are being developed for, and adapted accordingly.

The use of these criteria in the development of future bolt-ons may help to facilitate their approval for use in practice, which in turn could enable better estimates of HRQoL gains to be captured in health technology assessments. However, before that is possible, further research must be conducted to better understand how bolt-ons should be valued. The prospect of valuation is a fundamental feature in the development of EQ-5D items. The development of bolt-on items must consider the needs of valuation exercises. We have not explicitly specified criteria relating to valuation, but have noted where this is a relevant consideration.

The criteria that we have presented were identified as part of research to develop two new bolt-ons for the EQ-5D for vision and cognition using mixed methods. We have sought to describe generic criteria that we believe will be relevant to all future bolt-on development studies. However, given the complexity of health experiences, it is possible that they will not be appropriate in certain circumstances. Our recommended criteria should be seen as guidance and not as absolute requirements and can be adapted for the context and health area. Nevertheless, we encourage those developing bolt-ons to consider the criteria to guide their work.
